# Applying bioinformatics for antibody epitope prediction using affinity-selected mimotopes – relevance for vaccine design

**DOI:** 10.1186/1745-7580-6-S2-S6

**Published:** 2010-11-03

**Authors:** Galina F Denisova, Dimitri A Denisov, Jonathan L Bramson

**Affiliations:** 1 Department of Pathology and Molecular Medicine, Centre for Gene Therapeutics, McMaster University, 1200 Main Street West, Hamilton, Ontario, Canada, L8N 3Z5

## Abstract

To properly characterize protective polyclonal antibody responses, it is necessary to examine epitope specificity.  Most antibody epitopes are conformational in nature and, thus, cannot be identified using synthetic linear peptides.  Cyclic peptides can function as mimetics of conformational epitopes (termed *mimotopes*), thereby providing targets, which can be selected by immunoaffinity purification.  However, the management of large collections of random cyclic peptides is cumbersome.  Filamentous bacteriophage provides a useful scaffold for the expression of random peptides (termed *phage display*) facilitating both the production and manipulation of complex peptide libraries.  Immunoaffinity selection of phage displaying random cyclic peptides is an effective strategy for isolating mimotopes with specificity for a given antiserum.  Further epitope prediction based on mimotope sequence is not trivial since mimotopes generally display only small homologies with the target protein.  Large numbers of unique mimotopes are required to provide sufficient sequence coverage to elucidate the target epitope.  We have developed a method based on pattern recognition theory to deal with the complexity of large collections of conformational mimotopes.  The analysis consists of two phases: 1) The *learning phase* where a large collection of epitope-specific mimotopes is analyzed to identify epitope specific “signs” and 2) The *identification phase* where immunoaffinity-selected mimotopes are interrogated for the presence of the epitope specific “signs” and assigned to specific epitopes.  We are currently using computational methods to define epitope “signs” without the need for prior knowledge of specific mimotopes.  This technology provides an important tool for characterizing the breadth of antibody specificities within polyclonal antisera.

## Introduction

Antibodies play a central role in immune memory and long-term protective responses.  Serum antibodies for specific pathogens are recognized as a primary read-out for vaccination and recent studies have revealed that pathogen-specific antibodies persist for decades following vaccination [1].  It is important, however, to appreciate that not all antibodies can prevent infection.  As an example, a collection of monoclonal antibodies have been isolated against the West Nile virus envelope protein (E) which recognize distinct epitopes within the protein.  However, only antibodies that bind to specific epitopes can produce virus neutralization and protective immunity *in vivo *[2].  It was shown also that although many anti-HER2 antibodies inhibit the growth of cancer cells, some of them have no effect on cell growth, while others actively stimulate cancer growth.  It has been proposed that this wide spectrum of biological effects is related to the epitope specificity of the Abs and to consequent changes in receptor signalling [3][4].  Therefore, to properly characterize a protective humoral response and to use it for vaccine design, it is necessary to characterize the epitope specificity, in addition to antibody titers. 

A typical strategy for monitoring specific antibodies to known antigens involves the use of ELISAs coated with recombinant protein(s) or the vaccine itself.  When combined with serologic analysis of recombinant cDNA expression libraries (SEREX), ELISAs are a powerful tool for monitoring humoral responses in various disease states where the antigens may not be known *a priori *[5-7].  With regard to autoimmune antibodies, the availablity of whole transcriptome sequences and effective protein expression systems has made it possible to screen the complete human proteome.  Protein arrays can be used for monitoring responses to a broad range of proteins.  Such arrays have been produced using genomic information [6,7] or using information available in the SEREX database [8,9], which collects data for antigenic proteins identified using polyserum from cancer patients.  All of these methods are useful tools for measuring the magnitude and breadth of the humoral response, but they reveal little information regarding epitope specificity.  While it is technically feasible to engineer recombinant proteins with specific mutations designed to disrupt putative epitopes, due to the complexity of protein folding, epitopes can be disrupted at sites distal to the mutations that will complicate interpretation of the results.

Epitope-specificity can be determined for linear epitopes by screening libraries of short synthetic peptides that span the entire target antigen (PEPSCAN) [10,11].  However, the majority of antibody responses [12] are directed at structural epitopes which are difficult to recapitulate with synthetic peptides because they are typically formed by protein folding and, thus, are composed of amino acid residues which are often separated by great distances within the linear protein sequence.  Immunoaffinity selection of random peptides offers an alternate strategy to characterize antibody epitopes because the affinity selection will identify peptides with spatially-proximal residues that may be distant from each other according to linear sequence.  Indeed, this strategy offers an unbiased method to screen for epitope mimetics (mimotopes) [13] that can define antibody targets and serve directly as immunogens.  To effectively apply this strategy, it is necessary to isolate many random peptides with unique sequences because each immuno-selected peptide will carry only partial homology for the original target.  Through the use of computational modeling, it is possible to derive a consensus sequence from the selected peptides and identify the target epitope.  The use of linear random peptides for this strategy is limited because the conformation space available to linear peptides is great, allowing the linear peptides to assume a large array of conformations.  Constraining the peptide by cyclization reduces the field of conformational possibilities for the molecule and results in the peptide adopting the most favourable conformation [14].  Further, the constrained nature of these peptides causes them to adopt tertiary structure enabling them to mimic conformational epitopes.  In this way, cyclized peptides present a more uniform structure than linear peptides and, thus, are preferred ligands for mapping conformational epitopes.  Cyclization can be achieved simply by the incorporation of Cys residues at the N- and C-termini of a given peptide.   However, production and manipulation of synthetic random peptide libraries is costly and extremely laborious, necessitating the use of biological strategies to make it feasible.

### Genetic approaches for the production and manipulation of random peptide libraries

To facilitate the generation of random peptide libraries, genetic methods have been developed to introduce random peptides into scaffold proteins which can then be subjected to immunoaffinity selection followed by sequencing of the coding sequence of the random peptide.  Common genetic methods for displaying random peptide ligands including: phage display, bacterium and yeast display, ribosome display and mRNA display.  Incorporation of random peptides into scaffold proteins expressed on the surface of microbes (ex. phage display) are limited by the complexity of the sequence libraries that can be generated; for example, phage display libraries and bacterial display libraries are typically limited by transfection efficiency to approximately 10^9^  independent members [15-17].  The yeast, *Saccharomyces cerevisiae,* is very useful as a host cell in genetic engineering because it folds and glycosylates heterologous eukaryotic proteins [18] and can be used for surface display of eukaryotic proteins and peptides in a natural conformation [19]; although, this property is not advantageous for surface display of random cyclized peptides.  More recently, selection schemes based on the display of the nascent peptide chain on the surface of the ribosome have been developed [20-22]. This approach has the advantage of being fully *in vitro* and potentially allowing larger libraries (10^12^) to be explored; however, selections must be performed under conditions that preserve the integrity of the ribosome:mRNA:peptide ternary complex.  The ability to synthesize covalent mRNA-peptide fusions by *in vitro* translation provides a different approach to the *in vitro* selection and directed evolution of peptides and proteins. This approach should have significant advantages over all approaches that require an *in vivo* step, because libraries of much greater complexity can be generated *in vitro*. This is a critical advantage for experiments in which a rare functional sequence is being selected from a completely random sequence initial library. Current methodologies can yield mRNA-peptide fusion libraries consisting of 10^12^–10^13^ independent members [23,24].  However, the peptides expressed *in vitro* will not be reduced and, thus, cannot be used to generate cyclized ligands.  

Our work has focused on the use of phage display for the presentation of random cyclic peptides.  We employ the non-lytic phage, fd, that buds from their gram-negative E. coli hosts through the periplasm where disulfide bonds are formed due to the presence of the thiol-disulfide oxidoreductase (TDOR) family of enzymes [25].  By incorporating sequences encoding random peptides with only 2 Cys residues in frame with the N-terminus of the phage pVIII coat protein, we have produced libraries of cyclized random peptide with loops ranging from 4 to 12 residues in length.  Affinity-selection of phage displaying random cyclized peptides using specific monoclonal, or polyclonal, serum can yield mimetics of conformational and discontinuous antibody epitopes [26,27] as well as carbohydrate epitopes [28,29].  Computational modeling of the sequences of the immunoaffinity-selected mimotopes has been used with great success for the elucidation of target epitopes of monoclonal antibodies.  Improved transformation methods has enabled the production of highly complex libraries [30] making phage a desirable scaffold for the manipulation of highly-diverse libraries of cyclized  peptides.  Affinity-selection of specific mimotopes is typically accomplished by incubating the random peptide phage library with antibodies that have been immobilized on a solid matrix.  Iterative washing and binding steps allow for enrichment of phage carrying peptide inserts that are specific for the immobilized antibodies.  The selected peptide sequences are then analyzed and assigned a location on the target protein using algorithms that we designed based on specific correlation analysis [31,32] or a variety of other  algorithms that have been developed based on similar principles [33,34].

### Phage displayed mimotopes can be used to characterize antigen-specific polysera

In 1994, Folgori et al. employed a library of phage-displayed random peptides to characterize antibody specificities in polyserum from patients vaccinated with Hepatitis B virus surface antigen (HBsAg) [35]. They identified mimotopes of two different epitopes within HBsAg.  Sera from 20 different vaccinees displayed reactivity for these mimotopes, while sera of non-immune individuals failed to bind.  This study opened a new approach to diagnostics and vaccine development based on phage display epitope library screening.  Similarly, random peptide libraries were screened with polysera from patients with Lyme disease and 17 peptides were selected that distinguished patients with Lyme disease from healthy controls, demonstrating the value of this technology for developing serological diagnostic tests  [36].  

The phage display technology has been used to investigate the nature of anti-HIV antibodies present in polyserum from long-term non-progressors (LTNPs).  These individuals typically possess antibodies with broad neutralization [37-39].  Understanding the epitope targets of these broadly neutralizing antibodies could provide important information for developing diagnostic tools and vaccination strategies.  In the first study of this kind, Scala et al. identified ten mimotopes, which had wide cross-reactivity with polysera from LTNPs and SHIV-infected monkeys [40].  The phage-borne epitopes were immunogenic and four out of five monkeys immunized with these phages experienced lower levels of peak viremia following infection [40], supporting the use of this technology for vaccine development.  Another study was performed with polyserum from a single LTNP patient, which identified a single epitope located in gp41 protein.  Probing the isolated phages with variety of sera revealed that different sera have different affinity to mimotope variants (some sera did not bind some of the variants at all) thus demonstrating the diversity of the immune response [32].  This diversity likely reflects variability among the circulating virus strains within the regions that are targeted by antibodies; understanding the relationship between this diversity and effective neutralization will likely provide novel insight into the mechanisms of neutralization.  

Since mimotopes generally display only small homologies with the target protein, large numbers of unique mimotopes are required to properly analyze complex polysera.  The Dietrich group reported a study wherein ~700 mimotopes were selected by screening a phage display epitope library with eight LTNP patient sera [41].  Some of the mimotope sequences were attributed to HIV antigens based on linear homology and conformational similarities.  However, it remains unclear whether the predicted epitopes represent targets for neutralization since immunization of mice with mimotopes selected from their phage collection only produced modest HIV-specific antibody responses with low neutralizing potential.  Therefore, although this technology offers great promise for serological diagnostics, as in the case of Hepatitis B virus vaccinees and patients afflicted with Lyme disease, further refinement is required to employ this methodology for discerning specific neutralizing epitopes, as in the case of LTNPs. 

### Algorithms for deciphering epitopes using collections of affinity-selected mimotopes

In terms of characterizing polyserum using this methodology, our ultimate goal is to employ the mimotopes to define specific epitopes.  While this has been done successfully for monoclonal antibodies [11,32,42,43] where the target antigen is well-defined, this process is substantially more complicated in the case of polyclonal sera where a constellation of antigens are targeted.  A common strategy for epitope identification involves simple homology searches where the linear sequence of the peptide is aligned with the corresponding linear sequence of a putative antigen [41].  However, this approach is applicable only for linear, or partially-conformational epitopes, which consist of small linear fragments.  Strategies based on linear homology cannot be applied to genuine conformational epitopes since very few residues will be proximal to each other in the linear sequence.  Additionally, we have observed that application of the linear alignment method identifies short linear fragments (typically 3-5 amino acids) that appear to be specific but are actually non-specific as they can also be found in the irrelevant proteins that we use for negative controls (Denisov, Denisova and Bramson, unpublished data).  As an example, the sequence TPPG was uncovered as a common linear element within phage-displayed mimotopes isolated with polyserum from West Nile-infected individuals and this sequence maps equally well to great numbers of non-viral protein sequences taken from the NCBI protein database.  Similar results were obtained with other short homologies like PAS, RSLT and RRP.  The mimotopes containing these fragments can be attributed to many proteins suggesting that they likely represent common backbone motifs that provide a basic structure to the peptide for low-affinity antibody binding and they complicate unambiguous epitope mapping using linear homologies.  Similar observations have been made by other groups [44] where turn-like structures for the sequences DVQX, XPGS, DITX, and DXSF were conserved between specific epitopes and a number of unrelated proteins, suggesting that these linear epitopes have inherent conformational preferences.  

**Figure 1:**
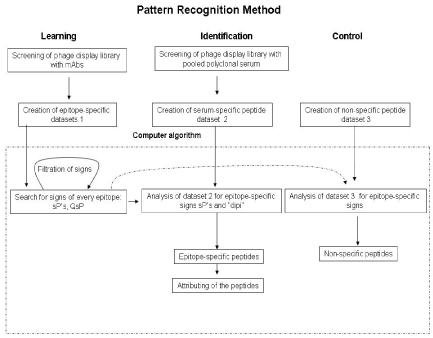
**Scheme of computer algorithm based on pattern recognition theory.** Signs – amino acid pairs chosen from different positions within an epitope sequence . Dipi – *discrimination parameter* equal to total number of epitope specific “signs” found at learning step. sP – “space pairs” -  amino acid pairs separated in a peptide by one, two, three amino acids . QsP - is the quality of sP  which is defined by the occurrence of the particular sP in all epitope mimotopes at learning.

To deal with the complexity of large collections of conformational mimotopes, we have developed a novel method based on pattern recognition theory [45-47]  (Fig. 1).  The analysis consists of two phases: learning and identification.  During the learning phase, a large collection of epitope-specific mimotopes is assembled through affinity-selection using specific monoclonal antibodies.  The collected peptides are analyzed to identify epitope specific *signs* using a novel computer algorithm that examines all possible amino acid pair combinations within the peptide collection and selects the combinations that are specific for the predicted epitopes.  The *signs* we employ are amino acid pairs chosen from different positions within an epitope sequence; unique signs can be identified which are specific to individual epitopes.   Such *signs* can also be identified by direct analysis of theoretically predicted epitopes [45,48].  During the identification phase, mimotopes selected using patient polyserum are interrogated for the presence of the epitope specific *signs* found during the learning phase.  Using statistical methods, mimotopes are subsequently identified based on their *signs* and assigned to specific epitopes.  We recently published a report describing the use of this approach for characterizing the epitope specificity of polysera from West Nile virus patients [45].  To accomplish this goal, we discovered *signs* during the learning step using a recently catalogued database of peptides that were affinity-selected using monoclonal antibodies specific for the West Nile virus E protein [31].  We then interrogated a collection of 106 peptides selected with West Nile virus-specific polyserum for evidence of epitope specific “signs” that were derived during the learning step.  Since many of these peptides contain common amino acid pairs, we also analyzed a similar sized collection of unrelated peptides to develop a threshold setting.  Implementation of this strategy is confounded by the complexity of human polyserum with regard to antibody diversity such that antibodies specific for a given epitope likely constitute only a small fraction of the total available antibody pool.  Therefore, it is expected that only a minority of the peptides selected by West Nile virus-specific polyserum can be attributed to epitopes on the West Nile virus E protein.  To select the most characteristic mimotopes for a given epitope, we defined a *discrimination parameter (dipi)* for every peptide which is equal to total number of epitope specific “signs” found at learning step and employed this parameter to establish the threshold.  The higher this threshold is set, the greater the likelihood that we will correctly attribute a given mimotope to the actual epitope.  However, the stringency provided by *dipi* must be balanced to permit selection of a sufficient number of peptides and to evaluate accuracy of the method.  Several peptides (10 of 29 in our case) contained signs that could be attributed to different antibody epitopes indicating that these peptides contain common motif.  To better understand the properties of these common motifs, we analyzed the total amino acid composition within the mimotope sets that were assigned to unique epitopes and those mimotopes that could be assigned to multiple epitopes. We found that the more ambiguous mimotopes were characterized by high proline content (22.86%) compared to the specific epitopes peptides (9.82%).  Proline is present at higher than expected abundance in random peptides presented by phage display [49,50].  Interestingly, proline-rich motifs have been implicated in multiple protein–protein interactions [51,52], so high proline content may reduce the need for specific interactions between the F(ab) portion of the antibody and the mimotope.  Thus, it seems likely that high proline content within certain mimotopes decreases the selectivity of our affinity-selection methodology.  However, we have overcome this issue by excluding peptides that are assigned to multiple epitopes with equivalent probability.  While such a filter will not completely remove nonspecific assignments, it does greatly reduce them. 

### Employing mimotopes as the basis for epitope-specific vaccination

While the mimotope strategy offers a powerful tool for monitoring epitope-specific antibodies following vaccination or infection, these mimetics may also provide a useful basis for the development of epitope-specific vaccines.  A critical parameter in this regard is the degree to which the mimotope accurately mimics the cognate epitope.  Some peptides selected from phage-display libraries by antibodies were used to successfully elicit cross-reactive antibody responses in animal models [53,54]; in some cases, these antibody responses have conferred neutralization *in vitro *[55] and protection [56].  However, many mimotope peptides failed to induce antibodies cross-reactive with corresponding antigens [57-60].  The issues related to the inability of mimotopes to elicit antibodies that were reactive to the native antigen were elegantly explored in the case of the B2.1 mimotope which was isolated using the anti-HIV neutralizing antibody, b12 [61,62].  Although B2.1 displayed high affinity for b12, immunization of mice and rabbits with B2.1 failed to elicit HIV-specific antibodies.  The crystal structure of b12 complexed with B2.1 was solved and compared to the structure of b12 bound to gp120 of HIV [61].  This comparison revealed that, although the binding in both cases was specific, the mimotope contacted different residues on b12 than gp120.  These data reveal that although affinity selected peptides are antibody-specific, they are not always true mimetics of the antigenic epitope.   While we, and others, commonly refer to immunoaffinity-selected peptides as mimotopes on the basis of their specific ability to bind antibody, it may be more appropriate to define them as binding site-specific peptides.   It is clear that some of these peptides can display high-affinity interactions with the antibody binding site in way that is distinct from the true epitope on the target protein.  The antibody binding site, which is formed by six hypervariable CDR loops, forms a continuous surface approximately 2800 A^2^ in area that is responsible for antigen binding according to X-ray crystallographic analysis.  Yet, only a fraction of the CDR surface is found to constitute the combining site.  As an example, for the monoclonal antibodies D1.3, HyHEL-5, HyHEL-lO, NC4I, and Je142, the surface that is used in the binding to antigen represents only 21%, 27%, 28%, 32%, and 22%, respectively, of the total surface formed by the CDRs.  Similarly, the CDR residues that contact the antigen represent only 25%, 37%, 36%, 33%, and 27%, respectively, of the total number of CDR residues.  It is not surprising, then, that when screening a complex library of random peptides, some peptides will be identified which bind to regions within the binding site that are not used to bind the target antigen.  In this respect, monoclonal antibodies can actually be considered polyspecific [63].  Indeed, screening of peptide libraries with a monoclonal antibody typically yields a collection of different peptides that all have high affinity binding [31,32,64-66].  Yet, upon simple examination, these peptides do not have significant similarity to each other despite the fact that they should share some of appropriately located contact residues to be able to bind the antibody.  Additionally, in view of the difference between sizes of epitope (average ~20 amino acids) and phage inserts, the mimotopes could mimic some aspects of epitope and different mimotopes could have partially overlapping sequences.  The second reason for inability of mimotopes to induce cross-reactive immune response could be difference in a shape of binding site of antibodies induced by native antigen and mimotopes. These shapes are determined by the shape of the antigen and it could be different for a flat protein surface and extended peptides [67].  During affinity maturation antibody binding site acquires the best complementarities for the peptide antigen and thus evolves from the best fit for protein antigen binding.  We believe that developing effective mimotope vaccines will require detailed structural analysis of the antibody binding to the target antigen to ensure that the best *mimetics* are selected in order to use them for the development of epitope-specific vaccines.

## Conclusion

Screening of a phage display library with polyclonal serum is technically simple and amenable to routine laboratory analysis.  This technology permits fine characterization of serum detecting not only antibodies directed to different domains of a protein but also antibodies directed to specific epitopes [68].  Using this methodology, it is possible to develop a characteristic “antibody signature” that can distinguish between different polysera directed at the same antigen [69].  Our results have demonstrated that the pattern recognition algorithm can effectively be employed to screen a mixture of antibodies and define the breadth of epitopes recognized by polyserum directed against specific proteins. The method can be further improved with a larger collection of mimotopes to be used in the learning step of the algorithm.  “Next Generation Sequencing” technology, which allows sequencing of one gigabase of DNA in a couple of days [70,71], might be beneficial in this regard, as it can enable, presumably, the sequencing of millions of phage in a single run.  In principle, our pattern recognition method could also be applied to cases when there are no available monoclonal antibodies by using computational strategies to predict antibody epitopes.  Multiple algorithms have been designed for prediction of antibody epitopes using atomic coordinates of the antigen or simple amino acid sequences [48][72-74].  For example, it is known that binding sites on a surface of proteins (commonly referred to as “hot spots”) have preferential amino acid composition, secondary structures and packing density.   Amino acids as tryptophan, arginine and tyrosine were shown to be enriched in these hot spots [75].   We used one of these approaches [73] for analysis of polyclonal immune response to HER-2 protein and found that our predictive algorithm gave the outcome similar to theoretically predicted epitopes [69]. Based on this information and the algorithm described in the current review, we are developing novel software tools that can predict all hypothetical protein antigenic sites and present as sets of amino acid pairs, which can subsequently be employed as epitope signs for use with our mimotope recognition computer algorithm.  

While the selected mimotopes may prove useful in the development of epitope-specific vaccines, the current state of the art still relies on a high degree of chance in the selection of the mimotope.  To facilitate mimotope selection for vaccine applications, highly diverse libraries should be used for screening of polyclonal neutralizing serum and large collections of mimotopes should be selected.  Computer assisted analysis of these large collections can help to select mimotopes enriched with epitope specific signs and scored according to the discrimination parameter.  Should this selection method fail to reproducibly identify useful mimetics, it may be necessary to combine our bioinformatics strategies with structural analysis to identify “best fit” peptides.

## List of abbreviations

HBsAg: Hepatitis B virus surface antigen; LTNP: long-term non-progressor; E protein: envelope protein; CDR: complementarity determining regions; Signs: amino acid pairs chosen from different positions within an epitope sequence; Dipi: *discrimination parameter* equal to total number of epitope specific “signs” found at learning step; sP: “space pairs” -  amino acid pairs separated in a peptide by one, two, three amino acids; QsP: the quality of sP  which is defined by the occurrence of the particular sP in all epitope mimotopes at learning.

## Competing interests

The authors cite no conflicts of interest.

## Authors’ contribution

All authors have equal contribution to this review.
